# Hydrogen sulphide exposure in waste water treatment

**DOI:** 10.1186/s12995-018-0191-z

**Published:** 2018-03-01

**Authors:** Åse Dalseth Austigard, Kristin Svendsen, Kari K. Heldal

**Affiliations:** 1 Municipality of Trondheim, Working Environment Office, Trondheim, Norway; 2 Institute of Industrial Economics and Technology Management, Norwegian University of Science and Technology, Alfred Getz vei 3, 7491 Trondheim, Norway; 3 The National Institute of Occupational Health, Oslo, Norway

**Keywords:** Peak exposure, Waste water workers, Hydrogen sulphide, Exposure index

## Abstract

**Background:**

The aims of this study was to assess exposure to hydrogen sulphide (H_2_S) among waste water treatment workers (WWWs), and achieve a better measure of the risks of H_2_S exposure than only using the eight-hour average value and the ceiling value because the exposure pattern of H_2_S for WWWs is dominated by short-term peaks.

**Methods:**

Ninety-three measurements of H_2_S from 56 WWWs in three cities and three rural areas were collected. All exposure measurements were carried out from the start of the day until lunch time (sampling time 4–5 h) when most of the practical work was performed. The type of tasks and extent of flushing were registered. H_2_S was measured using direct-reading instruments with logging: OdaLog L2/LL, Dräger X-am 5000 and Dräger Pac 7000 (0.1–200 ppm). Number and duration of peaks for different work tasks, seasons, places and extent of flushing were combined in an exposure index (IN), and evaluated in a mixed-model analysis, building a model aimed to predict exposure for different job tasks.

**Results:**

Nine Percent (8 of 93) of all H_2_S measurements have peaks above 10 ppm; in addition, 15% (14 of 93) have peaks of 5–10 ppm, 35% (33 of 93) have peaks of 1–5 ppm and 65% (62 of 93) have peaks of 0.1–1 ppm. 29% of the measurements of hydrogen sulphide showed no registered level > 0.1 ppm.

From the mixed-model analyses we see that exposure level, expressed as H_2_S index IN, varied between places, work type, season and degree of flushing. For the work in a plant in the capital, the exposure index varied from 0.02 for working in spring doing some flushing, to 0.7 for working at the same plant in winter doing flushing more than three times or more than 10 min. Collecting sewage from cesspools in city 2 in winter doing a lot of flushing gave a hydrogen sulphide index of 230.

**Conclusions:**

The use of a H_2_S index, taking into consideration peak height, duration and number of peaks, could be a tool for exposure assessment for H_2_S.

## Background

As a part of a project aiming to elucidate the association between respiratory and neurological symptoms and exposure in waste water treatment workers (WWWs), results from the measurements of hydrogen sulphide (H_2_S) when handling and transporting sewage in Norway are presented. To use the time weighted average (TWA) over one shift as a sole measure seemed inappropriate as one high peak could have health effects without being reflected by the TWA. However, no suggestions regarding what exposure measure having the most impact (peak level, peak frequency/number of peaks or peak duration) on health are reported in the literature.

WWWs commonly suffer from various health symptoms, including neurological and respiratory tract [1–5]. These effects have been ascribed to exposure to microorganisms and noxious gases such as H_2_S or from exposure to low levels of different biological and chemical agents [1, 4, 6].

In particular H_2_S exposure has been associated with cognitive impairment among WWWs [5] and neurological symptoms [2, 7].

H_2_S is a gas that can be produced by bacterial processes during the decay of both plant and animal protein or through the direct reduction of sulphate [8]. Therefore, it is a common pollutant in sewage plants and in agriculture as the process of cleaning the sewage needs the proteins to sediment from the water in which they are suspended. Further processing gives new possibilities for accumulation of H_2_S until the processed sediments are spread in the fields.

H_2_S can be extremely hazardous, being able to cause pulmonary oedema [9–11] sudden unconsciousness (> 500 ppm) or death by even a single exposure if the levels are over 1000 ppm [12, 13]. Repeated exposure to lower levels may result in more chronic respiratory symptoms [14] and symptoms from upper respiratory tract [15], the central nervous system, such as fatigue, headache, poor memory and dizziness [16]. As H_2_S exposure is characterised by short peaks, often with high intensity, it represents an extremely hazardous risk for workers because of the toxicity in high concentration combined with the pattern of exposure [1, 17]. Exposure levels are unpredictable, but the occurrence is associated with disturbance of biological material sedimented under lack of oxygen. The disturbance might be due to flushing of pipelines, movement in sediment basins, lowering of pressure on sediments or tidal water lifting sediments in overflow pipes. These are pipelines draining the pumping stations and the treatment plants, so they are not flooded during maintenance or extensive rain. This is necessary, as much of the sewage pipelines in Norway also handle surface water.

Sewage is treated to reduce pollution of rivers, lakes and fjords. This pollution might lead to the extreme growth of algae, sedimentation that uses all the oxygen in the waters and thereby kills bottom life. Sewage treatment also prevents spreading of diseases and other kinds of pollution thrown in the sewage. Treatment is based on biological degradation, physical sedimentation and/or chemical treatment to enhance sedimentation.

Studies of WWWs’ exposure to H_2_S are sparse. TWA exposure levels over work shifts have been reported to be low [1, 18]. TWA measurements over 8 hours have shown mean levels < 1 ppm [1, 3, 18] regardless of jobs. Other reported measurement levels are either stationary or task based [1, 3, 6, 19]. All these results show that eight-hour TWA is < 1 ppm. However, peak exposure above 100 ppm from stationary measurements during handling of sewage has been reported [1, 19]. At these levels, H_2_S does not smell, due to olfactory fatigue [20]. This means there is no smell to give exposure warning at levels of acute danger to life and health.

Several studies on exposure at low background levels of H_2_S showed no adverse respiratory or neurological health effects, but studies from Iceland showed that exceeding 7.00 μg/m^3^ (0.005 ppm) H_2_S were associated with increase in emergency hospital visits due to heart disease in elderly population [21].

Studies from the geothermal areal Rotorura in New Zealand with long term exposure levels of H_2_S at ~0.01–0.03 ppm provided no evidence of impairment of pulmonary function or with chronic obstructive pulmonary disease (COPD) [22].

The same authors studied H_2_S and self reported asthma and asthma symptoms without finding any increased asthma risk [23].

A study on inhabitants in the same area of H_2_S exposure and cognitive impairment found no association at these ambient levels [24].

Jäppinen [25] found that levels of 2 ppm H_2_S might cause bronchial obstruction in asthmatic individuals. In non-asthmatic individuals they could not find noticeable respiratory effects at levels below 10 ppm.

A review of H_2_S poisonings in the Alberta province of Canada over a 5 year period concluded that there were no long-term adverse effects apparent in the survivors. This conclusion was based on symptomatic description of the patients at their discharge from medical care as no follow-up tracings after discharge were available. Most of these incidences were related to the large Canadian oil industry [26].

As H_2_S is an acutely toxic gas that could rapidly reach harmful or even lethal levels, a short term exposure level (STEL) or a ceiling value is established in several countries. In Norway, this ceiling value is 10 ppm [27]. The no observed effect level (NOEL) for lesions in the olfactory mucosa after short-term exposure has been reported to be 10 ppm [28].

The aim of the present study was to map the exposure to H_2_S among sewer workers with regard to type of jobs, tasks, seasons and geographical place. In addition we wanted to suggest an index which may reflect another exposure measure than the TWA for H_2_S.

## Method

The study was conducted in two areas: around the capital and in five counties in the middle of Norway. Three big plants, all pump stations and the sewage network in the capital were included. In the middle of Norway, two cities and three rural counties were selected, with all their sewage work included. These counties were selected because of easy access and for their type of plants (the five biggest plants in Norway and one smaller, but still large plant) and because the plant sizes in the selected rural areas are found in smaller cities and all rural areas of Norway. All workers in the selected areas were invited to the study, and all participated, a total of 149 persons. H_2_S measurements were performed for those available on the measurement days, which gave a total of 56 different workers.

All types of sewage work executed by four separate groups of workers were included:

i. Big plants:
  daily inspection and cleaning activities. The inspection was mainly done from control rooms with automatic remote-control systems combined with visual control. The biggest city plant handled sewage from 600,000 person equivalents (PE) and the smallest city plant from 22,000 PE. The number of workers in each plant was from 4 to 20. The six plants were all located in rock halls and categorized as big plants.
ii. Pump stations and small plants:
  maintenance and cleaning of the pump stations and plants. This work includes flushing with water under high pressure (100–200 bar) and emptying of sediments from pits or storage areas.
  For rural workers, also working with the sewer network connected to the plant is included, as the work pattern is more complex and not easily separated during measurement.
  The plants in the rural areas handled sewage from 2000 PE to 5000 PE and employed from one to three workers.
iii. Sewer network
  This work includes flushing with water under high pressure (100–200 bar) and close contact with waste water.
iv. Collecting sewage from cesspools
  The sewage was removed using a tanker lorry equipped with a pumping device and unloaded from the tanker lorry at the plants or into sewer tunnels leading to the plants. Cesspool dimensions vary from approximately 1 m^3^ (one house) to over 20 m^3^. One lorry load is normally 3.5 m^3^. Intervals of emptying vary from 3 months (drinking water reservoir area) to 4 years (cottage, periodically habited). One and 2 years are the most common intervals. This work is not present in the capital.

Measurements of H_2_S exposure were performed on 56 of the workers, at different seasons and always included at least two different seasons for each city or area.

During sampling, the number of flushings was registered. The job types were categorized. Table 1 gives an overview of places and job types.

**Table 1:** The different cities and rural areas and the jobs and tasks measured.

Place	Type of plant and system	Type of jobs	Tasks
Capital	2 big plants, pump stations, sewer network	Working in big plant, Working with sewer network, Working at pump stations	Flushing and cleaning, maintenance, Inspection.
City 1	2 big plants, pump stations, sewer network, cesspools	Working in big plant, Working with sewer network, Working at pump stations, Collecting sewage from cesspools	Flushing and cleaning, maintenance, sucking sewage from cesspools
City 2	1 big plant, pump stations,1 small plant, cesspools	Working in big plant, Collecting sewage from cesspools, Working in small rural plants including sewer network and pump stations	Inspection, flushing and cleaning, maintenance, sucking sewage from cesspools
Rural 1	1 small plant, pump stations, sewer network	Working in small rural plants including sewer network and pump stations	Flushing and cleaning, maintenance.
Rural 2	1 small plant, pump stations, sewer network	Working in small rural plants including sewer network and pump stations	Flushing and cleaning, maintenance.
Rural 3	1 small plant, pump station, sewer network, cesspools	Collecting sewage from cesspools, Working in small rural plants including sewer network and pump stations	Flushing and cleaning, maintenance, sucking sewage from cesspools

### Exposure measurements

Ninety three personal measurements have been collected among WWWs, focusing on distributing the measurements over seasons, work task, persons, areas and size of cleaning facilities, all in an effort to explore the exposure and to suggest an exposure index that is better than the time weighted average (TWA) to evaluate the connection between exposure and health effects.

Our initial thesis was that because of the nature of the toxicity and exposure pattern of H_2_S, the levels could be expressed in an H_2_S index combining the characteristics of peak height, number of peaks and duration of peaks during the whole shift. The index is intended to be used for the analysis of H_2_S exposure and health effects in a coming study.

**Fig. 1:**
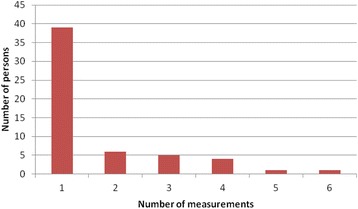
Number of measurement per person. Bar graph illustrating the number of measurements per person and corresponding number of persons

All exposure measurements were carried out from the start of the day until lunch time (sampling time 4–5 h) when most of the practical work was performed. Measurement time and time of active sewage work during measurement were registered. The workers carried a backpack, and the H_2_S sensors were fastened on the carrying straps on the front of their chests. The inlets of the sensors were in the breathing zone of the worker. Sampling were performed during the whole year and therefore classified in four seasons, winter (26 measurements, 10 days), spring (14 measurements, 4 days), summer (25 measurements, 11 days) and autumn (28 measurements, 8 days) measurements. Fifty six workers were measured, 30% more than once, and we obtained a total of 93 H_2_S measurements over a total of 33 different days. Distribution of measurements per worker is illustrated in Fig. 1.

H_2_S was measured with three different direct-reading logging instruments: OdaLog L2/LL, Dräger X-am 5000 and Dräger Pac 7000 (0.1–200 ppm; resolution 0.1 ppm). The instruments recorded 15 s concentration average, in a continuous log, four times per minute. H_2_S exposure was registered by the number of peaks measured within these intervals: 0.1–1.0 ppm, 1.1–5.0 ppm, 5.1–10.0 ppm and above 10.0 ppm. The total duration of peaks were noted and given for two intervals: 0.1–5.0 ppm and above 5.0 ppm. Therefore, to handle these measurement results as one value, a H_2_S index was suggested. We have thought of the index as dimensionless, even though it could be said to contain dimensions:

$$ {\displaystyle \begin{array}{l}{\mathrm{H}}_2\mathrm{S}\kern0.5em \mathrm{index}=\mathrm{IN}\\ {}\kern7.5em ={\mathrm{H}}_2{\mathrm{S}}_{01}\times 0.1+{\mathrm{H}}_2{\mathrm{S}}_{\mathrm{duration}01}\times 0.1+{\mathrm{H}}_2{\mathrm{S}}_1+{\mathrm{H}}_2{\mathrm{S}}_5\times {\mathrm{H}}_2{\mathrm{S}}_{\mathrm{duration}5}\times 5+{\mathrm{H}}_2{\mathrm{S}}_{10}\times 10+{\mathrm{H}}_2{\mathrm{S}}_{\mathrm{max}},\end{array}} $$

where.

H_2_S_01_represents the number of peaks in the lowest interval (0.1–1.0 ppm).

H_2_S_duration01_represents the time with level between 0.1 to 5 ppm(counted as number of minutes).

H_2_S_1_represents the number of peaks between 1.1–5.0 ppm.

H_2_S_5_represents the number of peaks between 5.1–10.0 ppm.

H_2_S_duration5_represents the time with level above 5.0 ppm (counted as number of minutes).

H_2_S_10_represents the number of peaks higher than 10.0 ppm.

H_2_S_max_represents the highest ppm level during measurement.

The values of number of peaks are multiplied with the set peak value: 0.1, 1, 5 or 10. This means that higher peaks are given more influence in the calculated index value when present.

Minutes is chosen as the time unit to be counted, as peak times are short. Most equipment can give values for such time, and it is easily counted from a graph or database.

This exposure index will be used as a determinant of exposure among others for different health effects in a later publication. The hypothesis is that not only average level but also the number of peaks and peak level are of relevance to health effects from the exposure. This is due to the behaviour of H_2_S, with peaking exposure as normal events, but peak levels are camouflaged if only the average is given.

### Statistical analysis

29% of the recordings gave H_2_S index values of 0 (0 peaks above 0.1 ppm). No H_2_S present is a rarity for this kind of work as long as active sewage work has been done, or deposits are present. Therefore IN = 0 was replaced with 0.04/√2 = 0.028, where 0.04 was 1/10 of the lowest calculated IN based on positive H_2_S recordings as a substitute for the detection limit [29]. This is also necessary due to the analysis methods; otherwise the number of measurements counted in statistical processes will be lower. This would increase the calculated uncertainty and give a wrong impression of the accuracy of the method.

The index was log transformed before statistical analysis, as workplace measurements are supposed to be log normally distributed. Logarithmical transformations give the possibility to check whether the data are consistent over magnitudes, and ease the mathematical calculations.

To build our model for predicting exposure in Table 4, the exposure to H_2_S, as H_2_S index (IN), was modelled by linear mixed-effect regression to account for the correlation between repeated measurements on the same person. By linear mixed-effect regression we can evaluate multiple parameters occurring during measurements. Determinants of exposure (job type, season, place and degree of flushing) were treated as fixed effects and worker as a random effect. The models were built stepwise by forward/backward selection. Determinants that significantly improved the models, judged by a *p*-value < 0.05 of the likelihood ratio test, were kept in the model. The effect of determinants is shown as factors by back-transformation of the log transformed regression coefficients. From the final model, exposure levels of different combinations of job type, season, place and degree of flushing, can be calculated as follows:

$$ \mathrm{E}={\mathrm{e}}^{\mathrm{c}+\mathrm{d}1+\mathrm{d}2\dots }={\mathrm{e}}^{\mathrm{c}}\times \mathrm{effect}\kern0.5em \mathrm{determinant}\kern0.5em 1\times \mathrm{effect}\kern0.5em \mathrm{determinant}\kern0.5em 2\dots, $$

where *E* is exposure as a calculated index for the specific set of determinants, *c* = intercept of the model and d1 and d2 are regression coefficients of determinants 1 and 2.

Arithmetic mean (AM) is a predictor of cumulative dose which is supposed to be the best exposure measure for long-term effects [30]. It can be calculated as

$$ \mathrm{AM}=\mathrm{GM}\times {e}^{\left(0.5\times \mathrm{variance}\right)} $$

where GM is the geometric mean, used for evaluating numbers that are log normally distributed, such as our H_2_S data.

“Variance” in this formula is the total variance. In our model this equals the calculated within worker variance (variance(ww)), because the between worker variance (variance(bw)) is < 0.00. This means that the day to day variation of a worker is higher than the variance between workers.

Having both the AM and GM gives an even better understanding of the distribution of data and gives a measure for the dose relevant to long term effects, which might also be necessary to give good advice on exposure protection.

Differences in H_2_S exposure as IN were analysed using one-way ANOVA with Bonferroni adjustments on the log transformed data, see Table 3.

All analyses were performed using IBM SPSS Statistics 20 (IBM, Armonk NY).

## Results

As the H_2_S peaks are normally steep, we have counted new peaks if the H_2_S level fell under detection limit, or if it has fallen to a lower category and then risen again. The highest number of peaks was 58. This was over a period of 160 min, where 130 of them were active in sewage work, and with 115 min recorded levels above 0.1 ppm.

Median number of peaks during measurements were 2, average were 7. Median time above 0.1 ppm for the measurements were 4 min, average 12 min.

**Fig. 2:**
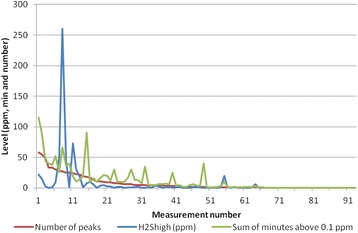
Peaks, their duration and maximum level. Number of peaks and their corresponding time above 0.1 ppm and maximum level of H_2_S

**Fig. 3:**
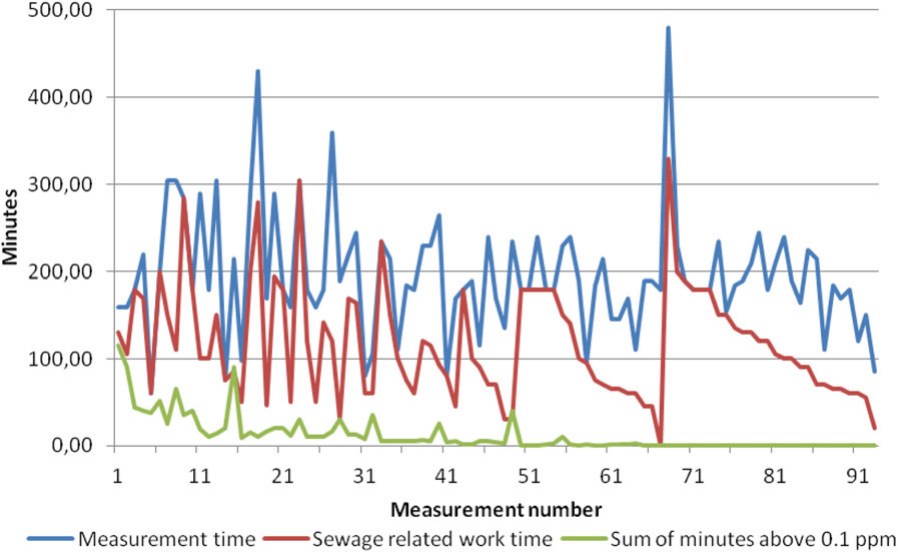
Measurement time and exposed time. Corresponding values for measurement time and exposed time, where “exposed time” is time with active sewage work. The measurements are sorted by number of peaks

Counting the peaks was a time consuming task, especially on the measurements with complicated (many) peaks, but mostly the peaks were easily counted. Graphical presentation of the corresponding numbers for minutes above 0.1 ppm, number of peaks, highest peak value, measurement time and time with exposed work in the measurements are given in Figs. 2 and 3. Measurement numbers correspond in the two figures. The measurements are sorted by number of peaks.

**Fig. 4:**
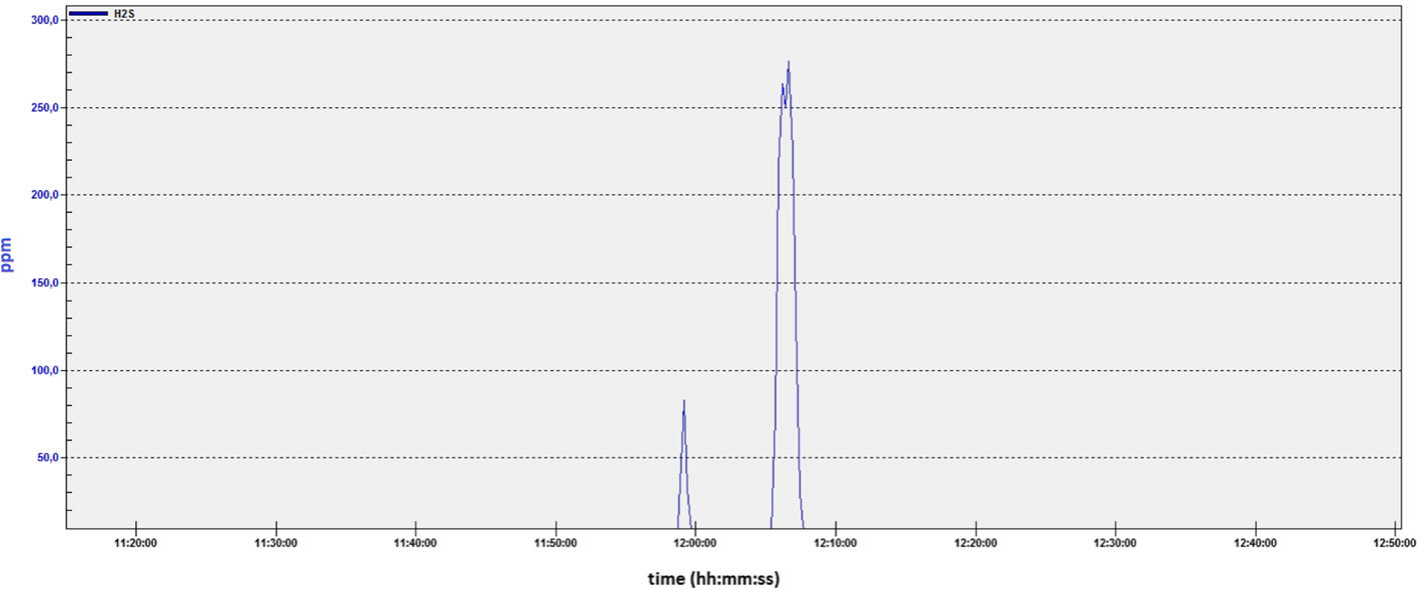
Example of measurement. Part of the highest exposed measurement

Part of the graph from our highest exposed measurement (Fig. 4) illustrates the problem with using average, or count of peaks or duration of peaks as single measure of this exposure. All these three measures would give “Low risk” as a predicted result. The peak height would of course give “High risk” in this measurement. When the same pattern repeats at lower levels this is not necessarily the case. The measuring range was 0.1–200 ppm, so the peak level might have been higher than registered. Normally the work also would have lasted longer, but was this time interrupted.

9% (8 of 93) of all H_2_S measurements have peaks above 10 ppm, in addition, 15% (14 of 93) have peaks of 5–10 ppm, 35% (33 of 93) have peaks of 1–5 ppm and 65% (62 of 93) have peaks of 0.1–1 ppm. 29% of the measurements of H_2_S showed no registered sulphide level > 0.1 ppm. Pump stations and small plants, work in sewer networks and collecting sewage from cesspools all had shifts with peak H_2_S above 10 ppm. The job with most excessive exposure to H_2_S was collecting sewage from cesspools, with 16% of all shifts having peaks above 10 ppm. During one shift, there could be as much as seven peaks above 10 ppm. The highest measured peak level (260 ppm) was also registered during this type of work. This was measured during collecting sewage that had been stored at a small plant for several months. The distribution of peaks for the different job types is given in Table 2.

**Table 2:** Measurement findings per category of plant and system

	Number of measurements (shifts)	Number of measurements with peaks (percentage of measurements) [average number of peaks in measurements with peaks]					Median time in minutes for the peaks above level (min-max)		Highest measured peak level in ppm
		> 10 ppm	5 ppm–10 ppm	1 ppm–5 ppm	0.1 ppm–1 ppm	Zero peaks	0.1 ppm	5 ppm	
Big plants	31	0	1 (3%) [2.0]	4 (13%) [2.5]	20 (65%) [5.2]	11 (35%)	5.5 (1–40)	2 (2)	10
Pump stations and small plants	25	3 (12%) [4.0]	5 (20%) [5.6]	16 (64%) [7.6]	20 (80%) [8.9]	5 (20%)	11 (3–90)	3 (1–25)	30
Sewer network	12	1 (8%) [1.0]	0	1 (8%) [1.0]	4 (33%) [3.0]	8 (67%)	15 (1–40)	5 (5)	20
Collecting sewage from cesspools	25	4 (16%) [3.8]	8 (32%) [2.3]	12 (48%) [4.0]	18 (72%) [6.8]	7 (28%)	7 (1–80)	5 (1–11)	260

The median, minimum and maximum H_2_S index for the different type of jobs, places, seasons and degree of flushing are given in Table 3.

**Table 3:** Descriptives for the different determinants of exposure expressed as hydrogen sulphide index (IN)

Determinants	Number of measurements (shifts)	Median	(min-max)
Job type:			
Inside big plants	31	0.7	(0–36.7)
Pump stations and small plants	25	5.5	(0–281.0)
Sewer network	12	0.5	(0–55.5)
Collecting sewage from cesspools	25	3.0	(0–338.6)
Time of the year			
Winter	26	3.4	(0–281.0)
Spring	14	0.7	(0–5.30)
Summer	25	6.3	(0–338.6)
Autumn	28	0.3	(0–55.5)
Place:			
Capital 1	11	0.9	(0–5.0)
Capital 2	11	1.0	(0–7.4)
Capital 3	14	0.4	(0–75.1)
City 1	25	0.7	(0–25.8)
City 2	15	4.4	(0–338.6)
Rural 1	2	2.8	(0–5.5)
Rural 2	8	8.4	(0–177.5)
Rural 3	7	13.1	(3.7–281.0)
Flushing			
No	34	0.6	(0–55.5)
Some	31	1.2	(0–25.8)
More than 3 times or over 10 min	28	7.6	(0–338.6)

The one-way ANOVA showed the following results regarding statistically significant differences (*p* < 0.05) between the categories for the predictors.

- Flushing: “More than 3 times or over 10 min” different from the two other categories
- Time of year: “summer” different from the two other categories
- Job type: “Inside big plants” different from “pump stations and small plants” and “collecting sewage from cesspools”
- Place: Rural3 is different from Capital1, Capital2, Capital3 and City1.
  City1 is different from Rural1, Rural2, Rural3.
  City2 is different from Capital1 and City1.

From the mixed-model analyses, we see that exposure level, expressed as H_2_S index, varied between places, work type, season and degree of flushing. For the work in a plant in the capital, the exposure index varied from 0.02 for working in spring doing some flushing, to 0.7 for working at the same plant in winter doing flushing more than three times or more than 10 min. Collecting sewage from cesspools in city 2 in winter doing a lot of flushing gave a H_2_S index of 230.

Table 4 shows the model with determinants from the mixed-model analysis.

**Table 4:** Linear mixed effect model of exposure determinants for hydrogen sulphide index (IN) in sewage workers

Factor	B	SE	e^B^
Intercept	5.02*	1.35	151.42
Job type:			
Inside big plants	−2.55	1.52	0.08
Pump stations and small plants	−1.73	1.51	0.18
Working on sewer network	0	0	1.00
Collecting sewage from cesspools	0.42	1.46	1.52
Time of the year:			
Winter	0	0	1.00
Spring	−2.49*	0.88	0.08
Summer	−1.10	0.82	0.33
Autumn	−1.36*	0.65	0.26
Place:			
Capital 1	−1.14	1.10	0.32
Capital 2	0.07	1.22	1.07
Capital 3	−2.80*	1.09	0.06
City 1	−2.07*	1.00	0.13
City 2	0	0	1.00
Rural 1	−1.90	1.33	0.15
Rural 2	−0.38	0.69	0.68
Rural 3	0.57	0.90	1.77
Flushing			
No	−1.10*	0.47	0.33
Some	−1.16*	0.45	0.31
More than 3 timers or over 10 min	0	0	1.00
Random effects	Variance		
Within worker	1.83		
Between worker	< 0.000		

*B* regression coefficient, *SE* Standard error, *e*^*B*^ Determinants for calculating GM for different operations of work

*p < 0.05

Example of calculation:

Geometric mean (GM) sulphide index of worker of pump stations and small plants in autumn, at place Rural 3 and some flushing then gives:

$$ {\displaystyle \begin{array}{l}\mathrm{IN}={\mathrm{Intercept}}^{\ast}\mathrm{Job}\kern0.5em {\mathrm{type}}^{\ast}\mathrm{Time}\kern0.5em \mathrm{of}\kern0.5em \mathrm{the}\kern0.5em {\mathrm{year}}^{\ast }{\mathrm{Place}}^{\ast}\mathrm{Flushing}\\ {}\kern2.5em =151.42\times 0.18\times 0.26\times 1.77\times 0.31\underline {\underline{=3.9}}\end{array}} $$

The arithmetic mean calculated for this work description is:

$$ \mathrm{Arithmetic}\kern0.5em \mathrm{mean}\kern0.5em \left(\mathrm{AM}\right)\kern0.5em \mathrm{sulphide}\kern0.5em \mathrm{index}=\mathrm{GM}\times {\mathrm{e}}^{\left(0.5\times \mathrm{variations}\right)}=3.9\times {\mathrm{e}}^{0.92}=3.9\times 2.51\underline {\underline{=9.8}} $$

We have calculated overall data such as GM, AM, minimum and maximum for the measured situations both from our linear mixed model and from our proposed index. These values are presented in Table 5 for the four types of job.

**Table 5:** Comparable calculations of IN from model and proposed index, based on measurement data and situations

Calculations of index IN	From Mixed model data				From Measurement data			
	GM	AM	Max	Min	GM	AM	Max	Min
Inside big plant	1.0	1.3	4.0	0.3	0,4	2.6	36.7	0.028
Pump stations and small plants	5.6	11.2	48.2	1.3	3,8	32.0	281.0	0.028
Sewer network	1.1	1.3	3.0	0.8	0.3	5.9	55.5	0.028
Collecting sewage from cesspools	3.8	19.6	76.0	0.7	2.2	41.4	338.6	0.028

**Fig. 5:**
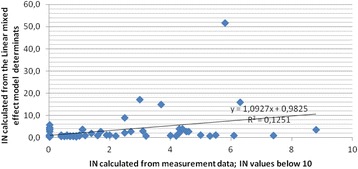
Model and measurement data. Fit-plot of IN values calculated from measurement data and IN values calculated from the mixed effect model data for identical situations

In Fig. 5, IN values from measurements are plotted against the corresponding IN value calculated from the model. It also show the regression calculated by excel of IN below 10 from the index. Almost 80% of our measurements are in this category. At higher values of IN from the index, the model data underestimates IN. This can be seen by comparing max-data from Table 5.

**Fig. 6:**
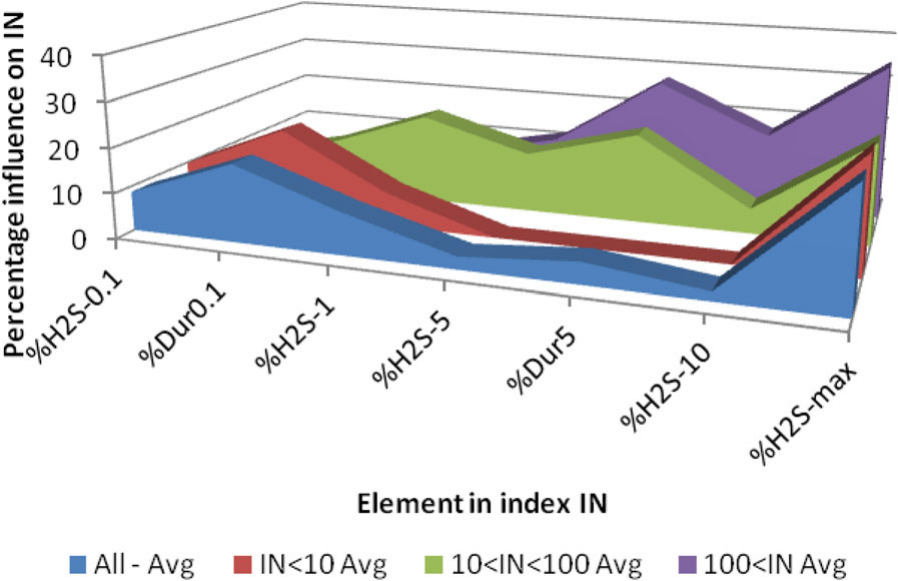
Contribution to IN. Distribution of percentage average contribution to IN for different intervals of IN

The influence of the different factors in IN varies with the level of IN. For levels below 10, obviously, the duration of peaks 0.1 ppm and the maximum level dominates. The dominating elements shift as the value of IN increases, and for values of IN> 100 number of and duration of the lowest peaks are negligible. The average percentage contributions for the different factors in different intervals of IN are shown in Fig. 6.

## Discussion

It was not surprising that collecting sewage from cesspools gave the highest IN (339), as most risks were present there: storage for several months (might be years) and often extensive flushing with high-pressure water to break the compact layer that covered the sewage. When breaking this layer, H_2_S was released from the sewer. The intensity of the H_2_S exposure was, however dependent on the time between emptying the cesspools. The measurement data from areas with frequent emptying (maximum one-year periods) did not have these peaks. The workers that collected sewage from frequently emptied cesspools did not need to use high-pressure water, but was able to suck up the sewage directly. There were no other noticeable differences between the cesspools. Frequent emptying of cesspools therefore seems important to avoid the use of high-pressure water (flushing), which easily releases H_2_S. This is shown in Table 4 of the mixed model as a factor 0.33 for no flushing and 1.00 for flushing more than 3 times per shift.

Another type of work that resulted in high peaks of H_2_S on a regular basis was maintenance on pump stations and small plants. The highest measured peak was 30 ppm and the highest number of peaks above 10 ppm in one shift was four (IN_max_ 281). They had more of the lower peaks and the lower peaks lasted for a longer time than cesspool emptying.

Other types of sewage work showed less frequent peaks above 10 ppm, but all types of jobs resulted in some H_2_S exposure. This H_2_S index also demonstrates that the degree of flushing has a great impact on the H_2_S exposure.

The different places of work give different exposure to H_2_S. This is partly because the different places had different work types. Some of the urban plants did not collect sewage from cesspools, nor did they have small plants to deal with. The work in rural areas, on the other hand, consisted of the two most exposed work types. This might have more to do with the technical standard than to size. As an example, all the big plants had ventilation from beneath covered sewage areas.

Our H_2_S index was constructed with weight on both low levels, duration and peak height because studies have indicated health effects from ambient air levels of H_2_S as low as 0.03 ppm [31] and at 0.3 ppm [16]. It seems, therefore, relevant to test a model that also emphasizes the lower exposure levels at 0.1 ppm. At this low level, the duration of exposure might have the same or higher significance than the level, as the documentation of health effects from these low levels originate from studies of ambient air concentrations measured over hours and days [16, 32–34]. However, in our IN model, these low levels of exposure were weighted less than higher levels.

As another set point for the duration, the Norwegian threshold limit value (TLV) at 5 ppm was chosen as this is the normal low alarm level for warning in personal alarm sensors worn by the WWWs. The weighting of our model was chosen in accordance with our equipment and the TLV and the STEL. The weighting of the different peaks and durations could, however, be changed. This index is no exact measure of the H_2_S level, but a relative estimate of exposure. By building an exposure model based on the index values of our measurements, we try to give a tool to estimate H_2_S exposure by task, place, season and extent of flushing.

When we treat the index as dimensionless, this is to simplify the use of the index and avoid misunderstandings with ppm values.

We chose to count number of minutes as our measure for time. It could be argued that we should have used 15 s periods, as this was our time resolution. One minute periods was chosen to ease the counting of our peaks. Improvement of the accuracy of the time could be done by counting number of resolution periods instead. If we then divide that number of periods by the number of periods in 1 minute, the values are still comparable to our index.

When using our H_2_S index for modelling exposure, we see that the exposure may vary with place. For city 2, where several work types were performed, the H_2_S index (geometric mean) varied between 0.3 for the workers that were inside the big plant when working in spring and doing no flushing, to 230 for those that collected sewage from cesspools in winter and doing flushing more than three times on the shift. The workers from city 1, also collecting sewage from cesspools, but without flushing with water (just sucking the sewage), had H_2_S index for winter of 30. This reflects the real difference between these two places. They performed a work operation that seemed to be the same, but the work was performed in two distinctly different ways, resulting in different exposure to H_2_S.

The detection limits in our measurements were 0.1 ppm. Our index may therefore not be valid for lower concentrations, and to use this index at lower values it must be given additional elements according to exposure profile.

Most of our measurements gave IN< 10, and all these measurements are below the Norwegian TLV and ceiling value. This is the normal exposure situation for WWWs in Norway. Almost 80% of our measurements are in this category. As shown in Fig. 5, there is a very good consistency between model values and index values of IN at values of IN below 10. Using the proposed index we can predict the normal exposure for WWWs quite well, but as the measurements show, there are regularly peaks and exposure duration that challenge the acceptable exposure. All values in our data set with IN> 100 have peaks above ceiling value. At such levels the model gives lower values than IN values from real measurements.

## Conclusion

This study shows that working with sewage often brings exposure to H_2_S. The exposure could have different patterns, and finding one measure for comparison between different tasks or workers could be challenging. The use of a H_2_S index, taking into consideration peak height, duration and number of peaks, could be a tool for exposure assessment for H_2_S. By using the model in sewage work in other places, IN can be predicted as an estimate.

## Abbreviations

AM: Arithmetic mean
COPD: Chronic obstructive pulmonary disease
GM: Geometric mean
H_2_S: Hydrogen sulphide
IN: Exposure index for H_2_S
m^3^: Cubic metres
NOEL: No observed effect level
PE: Eerson equivalents
ppm: Parts per million
s: Seconds
STEL: Short term exposure level. Normally 15 min average level
TLV: Threshold limit value. Normally 8 h average level
TWA: Time weighted average
WWWs: Waste water workers
